# Molecular taxonomical identification and phylogenetic relationships of some marine dominant algal species during red tide and harmful algal blooms along Egyptian coasts in the Alexandria region

**DOI:** 10.1007/s11356-022-19217-8

**Published:** 2022-03-14

**Authors:** Mona H. El-Hadary, Hosam E. Elsaied, Nehma M. Khalil, Samia K. Mikhail

**Affiliations:** 1 Department of Botany and Microbiology, Faculty of Science, Damanhour University, Damanhour, Al Beheria Governorate Egypt; 2 National Institutes of Oceanography and Fisheries (NIOF), Al kanater Elkhiria, Al Qalyubiyah Egypt; 3 National Institute of Oceanography and Fisheries (NIOF), Alexandria, Egypt

**Keywords:** HABs, Peroxidase isozymes, Protein profiling, 18S rDNA, Mediterranean Sea

## Abstract

Harmful algal blooms (HABs) threaten the aquatic ecosystems due to either poisonous effects on living organisms or oxygen-consuming. So HABs’ accurate identification, including red tide, is crucial. This study aimed to molecular identification of dominant species during tide period in nine stations along Alexandria region at Egyptian costs during one year. Samples were collected weekly before water discoloration but daily during red tide intensive growth from both 50 cm below the surface and 3 m depth over the bottom from the water surface. The red tide detection was highly from early August to half of September, since its highest peak with a maximum frequency inside the Eastern Harbor. The examined cultures samples isolated during red tide had four dominant species. Peroxidase profile showed an expression pattern of three loci (Px1, Px2, and Px3) in most species. The Px2 was the only heterozygous locus among the three loci in all species. Protein profiling showed that 17 bands out of 65 were specific to the species. The phylogenetic relationships derived from profiles of protein and 18S rRNA gene operon sequences for the four isolated species were mostly similar. We identified the four dominant HABs species as *Aplanochytrium* sp., *Chlamydomonas* sp., *Cryptophyceae* sp., and *Psammodictyon* sp. based on their 18S rRNA sequences and deposited them at DDBJ/EMBL/GenBank database. *Aplanochytrium* sp. is recorded as a red tide causative species for the first time in the screened region despite belonging to the defunct fungi.

**Supplementary Information:**

The online version contains supplementary material available at 10.1007/s11356-022-19217-8.

## Introduction

Harmful algal bloom (HAB) is a serious widespread environmental problem in coastal regions and semi-enclosed water areas. This phenomenon harms aquatic ecosystems and arises from both slow water movement stream and hyper-eutrophication by human activities (Luckas et al. 2005; Brooks et al. 2015). HABs have been observed since the 1930s in the coastal waters of many countries, including Japan, Norway, Ireland, New Zealand, and China (Wang et al. 2014). HABs are frequent in the USA (Anderson et al. 2021), Northeastern Arabian Sea, the coastal Red Sea (Mohamed 2018), and the Mediterranean Sea (Zingone et al. 2021).

The HAB has hazardous effects on aquaculture, fisheries, swimming activities, human health, and companion animals (Wang et al. 2014; Brooks et al. 2015). The HABs cause dissolved oxygen reduction and algal toxin elevation in the aquatic ecosystem food chain (Luckas et al. 2005; Reddy and Mastan 2011; Abou El-Geit et al. 2013). The most common HABs whose bloom discolors water with red color when the causative species are dinoflagellates (Wang et al. 2014; Zingone et al. 2021). In addition to the red tide by dinoflagellates, there are other types of discolors: the water with brown, pink, orange, yellow, green, or white colors. Water discoloration depends on the phytoplankton type to whom the causative species belong: dinoflagellates, diatoms, or blue-green algae (Ibrahim 2007; Reddy and Mastan 2011).

The HABs involve a wide range of species from different taxonomical levels. Biochemical and molecular probes can provide accurate identification for different taxa. The genetic variations detection can solve the potential collapses among HAB morpho-species, unlike morphological identification (Anderson et al*.* 2012). Biochemical markers such as SDS-PAGE enable following the seasonal variations in protein patterns of red algae (Rouxel et al*.* 2001). Application of PAGE analysis to differentiate between taxonomically confused strains of a single species could be a powerful taxonomic tool (Chan et al. 2002, 2004). The SDS-PAGE is a tool for characterizing toxic and non-toxic strains of different plankton genera (Lyra et al. 1997; Jiang et al. 2015; Li et al. 2019). Consequently, SDS-PAGE enables rapid identification by driving phylogenetic analysis based on genetic information and morphology (Lee et al*.* 2015). Also, the isozyme profile is an efficient implementation for detecting inter- and intra-species genetic variation among species (Micales et al. 1992; Saini and Yadav 2014). DNA probing and biochemical methods have a great efficiency in monitoring and taxonomy of HABs (Hallegraeff (1993); Hallegraeff et al. 2003; Anderson et al. 2012). Sequence analysis of genes including nuclear DNA, DNA plastid, large ribosomal subunit domains (LSU rDNA), small-subunit ribosomal (SSU rDNA), internal transcribed spacer sequence (ITS), and in situ hybridization (ISH) are reliable molecular tools for species identification (Damare and Raghukumar 2010). Gene sequence analysis provides the opportunity to reconstruct phylogenetic relationships among HAB taxa (Anderson et al. 2012) such as the case of dinoflagellate (Hong et al. 2008). For instance, the LSU domains of D1–D3 rDNA enabled molecular identification of *Heterocapsa* sp. and denied belonging to the monophyletic group that was endorsed by its morphological characteristics (Fariman and Javid 2013). The LSU rRNA-targeted oligonucleotide probes based on hybridization detected the fragile species *Heterosigma akashiwo* and *Fibrocapsa japonica* (Raphidophyceae) (Tyrrell et al. 2004). Species-specific sandwich hybridization assays were successfully developed for various raphidophytes (Tyrrell et al. 2004). Khaw et al. (2020) reported 18S rDNA priming as a simple method for cultured eukaryotic microalgae identification. Also, mitochondrial and chloroplast genomes were efficient in phylogenetic analyses and comparative genomic analyses of coastal diatoms (Liu et al. 2021a, b). Kobayashi et al. (2014) referred to the multiple sequence alignment of heat shock protein and the phylogenetic tree of some algae as well as their protein and cDNA expression patterns determining the survival threshold temperature in *Cyanidioschyzon merolae* and *Chlamydomonas reinhardtii*. Species-specific differences between the two species *Ochromonas* sp. and *Dinobryon* divergence were identified by dominant SSU rRNA genotypes (Auinger et al. 2008). The SSU sequence compared with ITS1 and ITS2 regions (including the 5.8S) of the ribosomal operon confirmed the identification of UWO 241 strain as *Chlamydomonas raudensis* Ettl and contradicts the previous designation as *C. subcaudata* Wille (Pocock et al. 2004). The ITS sequences of the rRNA gene succeeded in *Heterosigma akashiwo* Hada identification (O’Halloran et al. 2006). The sequence of 16S rDNA of the plastid evaluated the expression level of photosynthesis genes in some species (Mortazavi et al. 2008; Dierssen et al. 2015).

The HABs species detection in coastal regions is crucial for controlling the phenomena and avoiding dramatic events on human activities and aquatic life. The monitoring and management of HABs requires accurate information on the scale and nature of the problem, and efforts are needed to expand and sustain the collection of data regionally and nationally (Anderson et al. 2021). Harmless/harmful bloom was detected in Eastern Harbor (EH) due to favorable seasonal changes and eutrophication by wastewater and harbor identity as a semi-enclosed marine basin in the Alexandria region north Egypt. HABs causative species at the harbor are brought from different parts of the world across the overloaded bodies of giant ships during their journey. Ballast tank drainage for balancing giant ships, gods, the received water streams, and rainwater drainage in the harbor is well definite overloaded sources for HABs. However, there are no notable human extra activities at the EH. Morphological identification of commonly red tide causative species in EH pointed pertinence to either dinoflagellates or raphidophytes (Labib. 2002; Mikhail and Labib 2012).

The objective of this study is isolating and detecting molecular identification with 18S rDNA sequencing and phylogenetic reconstruction for the four HABs during red tide along with Egyptian costs at the Mediterranean Sea in Alexandria region including EH. The study offered molecularly identified database inputs for the dominant HABs species during red tide for the first time in Alexandria waters.

## Materials and methods

### Sampling collection stations and conditions

**Fig. 1:**
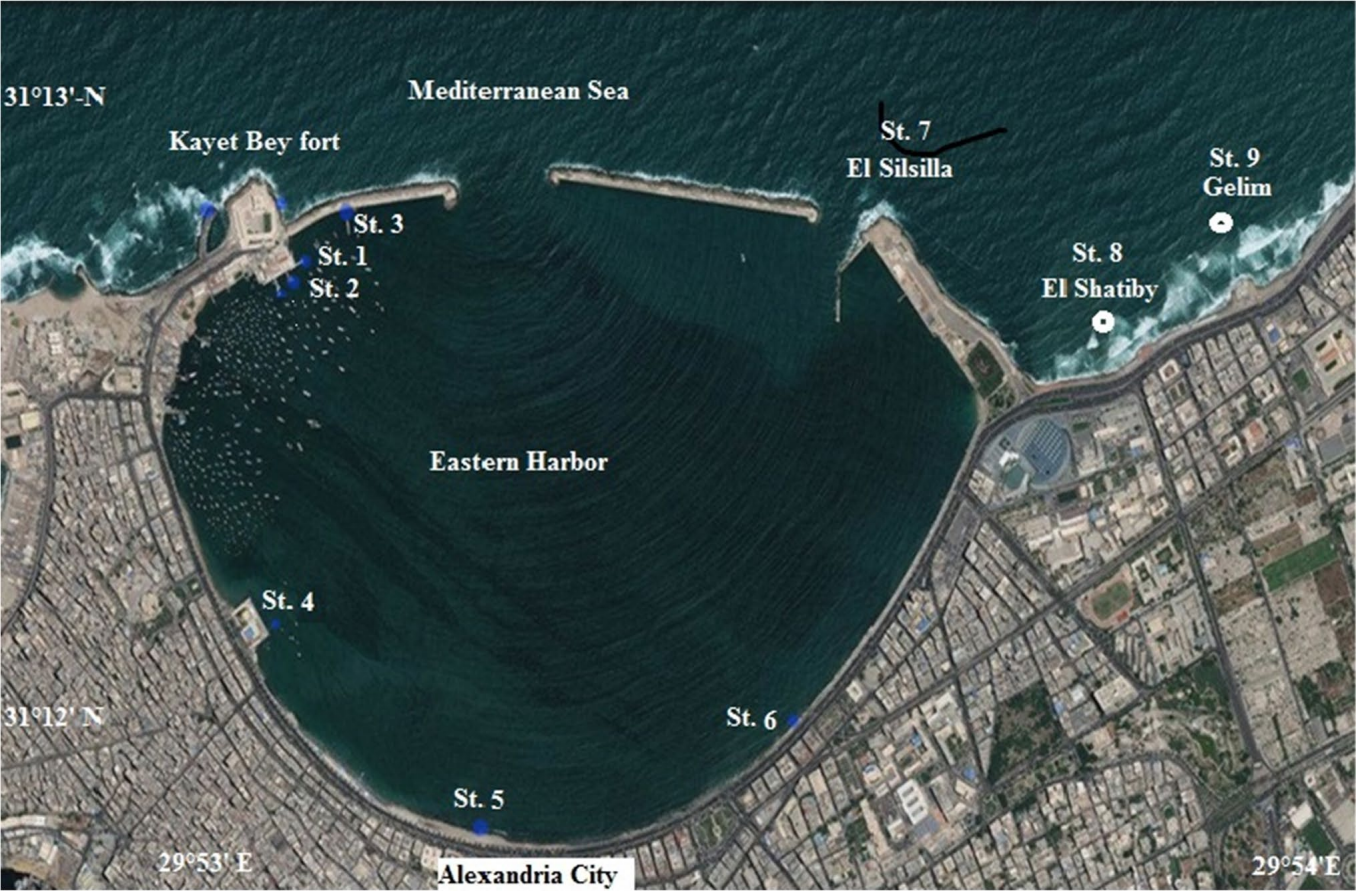
Sampling stations and Eastern Harbor location in Alexandria north Egypt, St. 1~9 indicating the station

The samples collection was from nine stations along the Alexandria region on the Egyptian costs (Fig. 1) throughout a year cycle (March 2015–March 2016). Six stations (St.1~6) out of nine were inside the EH that is a semi-enclosed marine basin (longitudes 29°53′–29°54′-E and latitudes 31°12′–31°13′-N with an area of about 2.53 km^2^, average depth about 6.5 m, and water volume of 15.2 × 106 m^3^). The other three stations (St. 7~9) were outside the harbor, known as El-Silsila, El-Shatby, and Gleim. The samples were collected directly from a depth 50 cm below the water surface (using a 2-L bottle) and 3 m over the bottom (using a water sampler).

Station 1 (St. 1) was a fixed point for following up HABs weekly. The daily sampling for the mentioned nine stations was syntonic to dense water discoloration appearance when reached maximum inside the harbor. The intensive red tide period was between early August and mid-September when the range of the temperature was 30.4 to 32.9 °C and pH was 8.3 to 8.8. Twenty litters of water samples were filtered through plankton net (mesh size 20 μm.). The samples were first examined to identify living flagellates and then preserved by the addition of 4% neutral formalin and a few drops of Lugol^’^s solution (Throndsen 1978). The species were investigated by the light inverted microscope, and the phytoplankton cells were countered according to Utermöhl (1958) as a unit per litter (units L^-1^).

### Samples culturing

The isolated cultures collected during red tide periods were grown at 25 °C under a 12:12 h light/dark cycle, in a filtered autoclaved seawater with Guillard’s f/2 marine water enrichment solution (1x Sigma G9903) as was described by Guillard and Ryther (1962) as was shown in Table 1. For obtaining pure cultures of individual dominant species, the collected samples were sub-cultured many times. The four dominant species were detected, morphologically identified, cultured, and symbolized as Euk-EH1, Euk-EH2, Euk-EH3, and Euk-EH4.

**Table 1:** Composition and preparation of Guillard’s F/_2_ medium Guillard and Ryther (1962)

Nutrients	Final concentration (mg.l^-1^ seawater)^a^	*Stock solution preparations
NaNO_3_	75	Nitrate/phosphate solution Working Stock: add 75 g NaNO_3_ + 5 g NaH_2_PO_4_ to 1 liter distilled water (DW)
NaH_2_PO_4_.H_2_O	5	
Na_2_SiO_3_.9H_2_O	30	Silicate solution Working stock: add 30 g Na_2_SiO_3_ to 1 liter DW
Na_2_C_10_H_14_O_8_N_2_.H_2_O (Na_2_EDTA)	4.36	Trace metal/EDTA solution Primary stocks: make 5 separate
CoCl_2_.6H_2_O	0.01	1Liter stocks of (g.l^-1^ DW) 10.0 g CoCl_2_, 9.8 g
CuSO_4_.5H_2_O	0.01	CuSO_4_, 180 g MnCl_2_, 6.3 g Na_2_MoO_4_, 22.0 g ZnSO_4_
FeCl_3_.6H_2_O	3.15	
MnCl_2_.4H_2_O	0.18	Working stock: add 1 ml of each primary stock solution + 4.35 g Na_2_C_10_H_14_O_8_N_2_ + 3.15 g FeCl_3_ to 1 liter DW
Na_2_MoO_4_.2H_2_O	0.006	
ZnSO_4_.7H_2_O	0.022	
Thiamin HCl	0.1	Thitamin solution (t x) Primary stock: add 20 g thiamin HCl + 0.1 g biotin + 0.1 g B_12_ to 1 liter DW
Biotin	0.0005	
B_12_	0.0005	Working stock: add 5 ml primary stock to 1 liter DW

*Store all stock solutions in the refrigerator

The dominant HAB isolated species were constrained to the following investigation.

### Protein profiling

The water-soluble protein of the four cultured species was extracted by grinding 1 g biomass of each sample in 1 ml extraction buffer and then vortexed for 2 min by the laboratory mini centrifuge/bench-top with vortex shaker, FVL-2400- BOECO, Germany. Samples were shacked for 2 h and then centrifuged for 15 min at 14000 rpm at 4°C in the High Speed Table Top Refrigerated Centrifuge-HERMLE Labortechnik GmbH-Wehingen, Germany. The isolated protein supernatant was quantified by Bradford (1976) before fragmentation on 15% SDS-polyacrylamide gel electrophoresis (SDS-PAGE), according to Laemmli (1970). A sample volume of 30 μg protein concentration in a 20 μL loading buffer was heated in a water bath to 65 °C for 5 min and then cooled suddenly. The samples were loaded against a protein marker onto the stacking gel under 75 volts which elevated to 125 volts for approximately 2 h for resolving gel at Omni PAGE 2-D Mini Systems Gel Electrophoresis Model CVS 10C2DSys, Cleaver Scientific UK. The gel was stained with 0.1 % Coomassie blue R-250 for 2 h before distaining. The developed protein banding pattern was documented by a gel documentation system (Molecular Imager^®^ Gel Doc™ XR, Bio-Rad, UK).

### Peroxidase isozymes profiling

A native-polyacrylamide gel electrophoresis (native-PAGE) of 10% was carried out according to Laemmli (1970). The enzyme was isolated using the method of Stegmann et al. (1985). Each isolated precipitated culture was re-suspended in 2.5 ml Tris-borate extraction buffer (0.125 M, pH 8.9) and kept shaking at -4 ^°^C for 2 h in the High Speed Table Top Refrigerated Centrifuge-HERMLE Labortechnik GmbH-Wehingen, Germany. The suspension was centrifuged at 1200 rpm for 10 min filtered, quantified, and used for isozyme profiling. Aliquots volumes of 20 μL for each sample were loaded onto gel slots against a slot containing bromophenol blue as marker dye at Omni PAGE 2-D Mini Systems Gel Electrophoresis Model CVS 10C2DSys, Cleaver Scientific, UK. Electrophoresis was run at 100 V for 3 h. The gel submerged in a reaction mixture of pH to 5.0 (0.25 g Benzidine dihydrochloride, four drops Glacial acetic acid, 100 ml distilled H_2_O) was incubated with ten drops of 1% freshly prepared hydrogen peroxide till developing of bands. For developing the isozyme pattern, the gel was transferred to distilled water. Then, the developed pattern was documented by the gel documentation system (Molecular Imager^®^ Gel Doc™ XR, Bio-Rad, UK).

### Sequencing of 18S rRNA operon

For DNA sequencing, the DNA was extracted and purified, using under sterilized conditions in JSR JSCB-1200SB Biohazard Safety Cabinet- Korea, using autoclaved tools (MC-30L Japan Autoclave). The isolated four species were preserved with Lugol’s solution. Stored isolates were filtrated using GV. 0.22 μm membranes filter Millipore, washed in a filtration system with Tris-EDTA (TE) buffer. The isolates hung with the filter paper are processed into DNA isolation steps, using a DNA isolation kit (MO BIO Laboratories, 12888-50, Carlsbad, CA) according to the manufacture’s protocol with some modifications of Elsaied et al*.* (2002) method. The extracted DNA was purified and the integrity was assured at 0.9% agarose gel at MultiSUB Choice, Wide Midi Horizontal Electrophoresis System, Cleaver, UK. The developed electrophoresed gel was UV visualized and documented by the Molecular Imager^®^ Gel Doc™ XR, Bio-Rad, UK.

The 18S rDNA was amplified using the primers Eukarya Forward 5′-ACG CTT GTC TCA AAG ATT A-3′ and Reverse 5′-ACGGAAACCTTGTTACGA-3′ (Lepere et al. 2011). The PCR reactions were performed by using a ProFlex™ PCR System Applied Biosystems (Life Technology, Thermo Fisher Scientific, USA) under reaction conditions of 1 cycle 95 ^°^C/3 min for initial denaturation, 96 ^°^C/ 50 s for denaturation, 12 cycle of 55 ^°^C (- 0.1 ^°^C every 1 cycle)/50 s for annealing, 72 ^°^C/1 min for extension, 72 ^°^C/12 min for final extension, and 1 cycle/∞ for final holding. The specimen was amplified against negative control and positive control of known DNA using a volume of 50 μl PCR reaction mixture. The reaction mixture was composed of 5 μL of 10x EX-Taq buffer (Mg2+ free), 5 μL of 2.5 mM for each dNTPs, 5 μL of 25 mM MgCl2, 0.3 μL of 250 U Takara EX-Taq™ Polymerase, 2 μL of 0.25 μM for both forward and reverse primers and 0.8 μL of 500 ng DNA template for sample or positive control. Amplicons were loaded to 1.24% agarose gel electrophoresis followed by staining with 20 μL/100 mL ethidium bromide (10 mg/mL) and visualized by UV-Molecular Imager^®^ Gel Doc™ XR, Bio-Rad, UK. The amplicon-specific bands were excised from the gel with a flame-sterilized scalpel.

DNA was extracted from the gel slices using a DNA gel elution kit (Catalog no. 42600, Amicon, Millipore). The PCR-amplicons quality, of the amplified DNAs for 18S rRNA, was tested by running on 1.5% agarose gel electrophoresis, followed by staining with ethidium bromide (EtBr) and UV visualization by Molecular Imager^®^ Gel Doc™ XR, Bio-Rad, UK.

The PCR amplicons were undergone sequencing analyses using Applied Biosystems Veriti® thermal cycler, Thermo Fisher Scientific, USA. The sequencing reaction was run with BigDye® terminator cycle sequencing kit, according to the manufacturer’s protocol (Applied Biosystems, Foster City, CA conditions). A volume of 10 μL DNA sequencing reaction composed of 2 μL BigDye® terminator, 2 μL 5x BigDye® buffer, 1 μL forward primer (3.2 pM), 0.8 μL DNA template (20 ng/μL), and 5 μL H_2_O (nuclease free) was used. Reaction conditions was 1 cycle 95 ^°^C/3 min for initial denaturation, 96 ^°^C/50 s for denaturation, 12 cycle of 55 ^°^C (- 0.1 ^°^C every 1 cycle)/50 s for annealing, 72 ^°^C/1 min for extension, 72 ^°^C/12 min for final extension, and 1 cycle/∞ for final holding.

Sequencing products were purified using the BigDye XTerminator® purification kit according to the manufacturer’s protocol (Applied Biosystems, Foster City, CA) in reaction mixture volume of 65 μL containing10 μL PCR solutions, 45 μL SAM^TM^ solution, and 10 μL XTerminator® solution. The amplicons were run on a capillary sequencer (Applied Biosystems Hitachi 3500 Genetic analyzer sequencer, Life Technology, Thermo Fisher Scientific, USA) for 3 h.

### Bioinformatics data analysis

Gels profile of protein and peroxidase were documented with UVP gel documentation system, model, GelDoc-It, England. The data analysis was carried out by using total lab analysis software, ww.totallab.com (Ver.1.0.1). The genetic distance was computed by Past software analysis (http://nhm2.uio.no/norlex/past/download.html), and the genetic trees were constructed. Polymorphism calculations depended on a zero/one matrix for the profiling pattern of both protein and isozymes. The polymorphism percentage is calculated as

$$\%\mathrm{Polymorphism}=\frac{\mathrm{Polymorphic}\ \mathrm{DNA}\ \mathrm{fragments}\ \mathrm{x}100}{\mathrm{Total}\ \mathrm{number}\ \mathrm{of}\ \mathrm{loci}}$$

DNA sequences of the four isolates were aligned by FASTA to determine their similarity to the known sequences in the DNA database for 18S rRNA (http://www.ebi.ac.uk/Tools/sss/fasta/), and the phylogenetic trees were constructed.

Construction of the phylogenetic trees was done through two bioinformatics programs. The phylogenetic tree recruited both the targeted rRNA gene phylotypes sequences and their homologous sequences obtained from the DNA database, beside out-group sequences aligned by using the online program “Clustal Omega” software (http://www.ebi.ac.uk/Tools/msa/clustalo/). Secondly, the phylogenetic tree involved the submission of the aligned sequences to MEGA 6.06 software (http://www.megasoftware.net/), for the construction of consensus phylogenetic trees, using maximum likelihood, neighbor-joining, and maximum parsimony algorithms, located in the same software. Bootstrap values were provided, as a phylogeny test, using 500 bootstrap replications.

The obtained sequences were deposited in the international DNA database of the Bank of Japan; DDBJ/EMBL/GenBank database (https://www.ddbj.nig.ac.jp/ddbj/updt-e.html ) under accessions numbers of LC377045, LC377046, LC377047, and LC377048 for the four studied phylotypes.

## Results

### Protein pattern

**Fig. 2:**
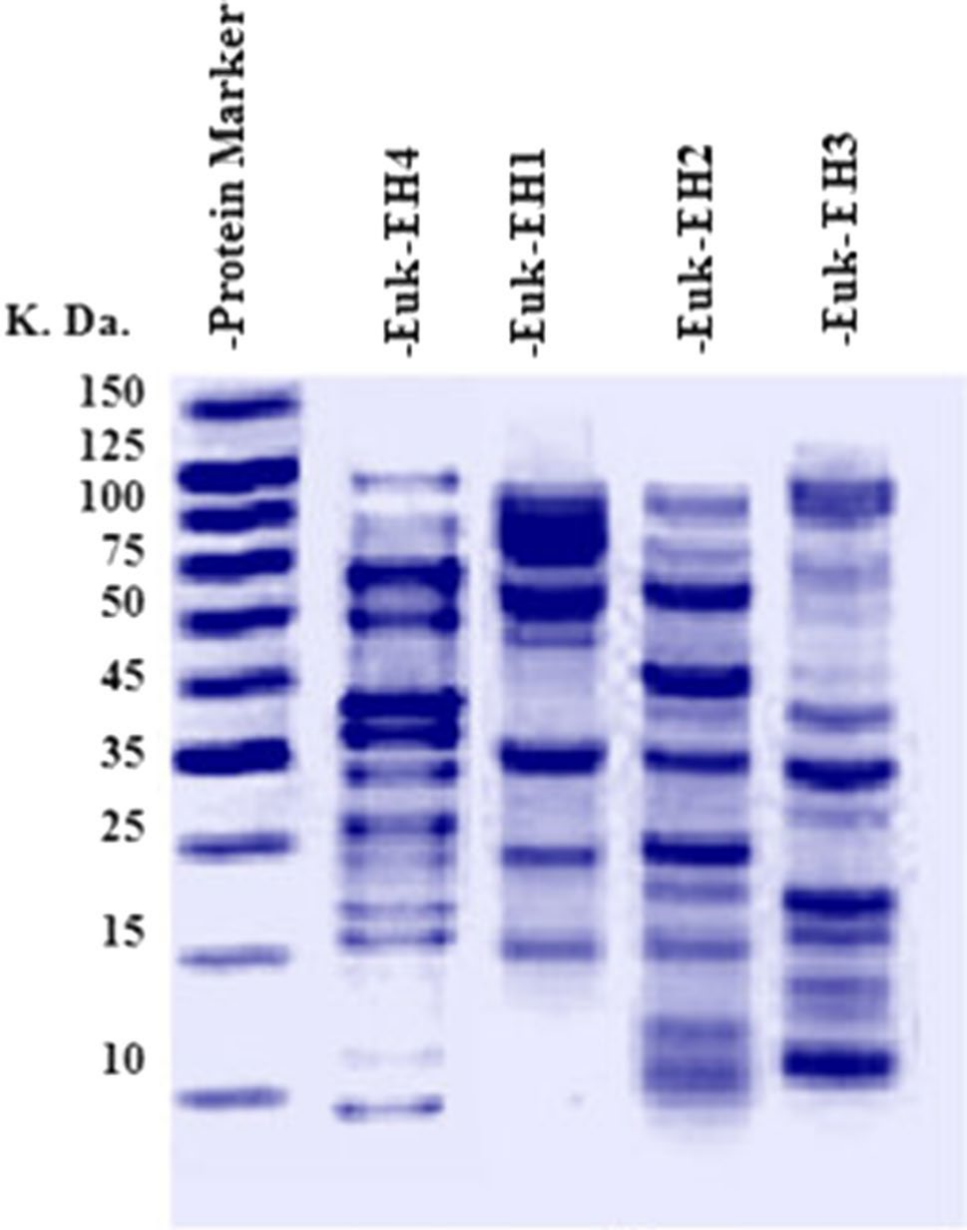
Protein fingerprinting patterns for the four dominant species during red tide in Eastern Harbor

Protein fingerprinting of the four dominant HABs species developed a pattern of 65 protein fraction bands in total, ranging from 9 to 120 KDa (Fig. 2). The species had differential 17 specific protein fraction bands out of the total pattern (Table SI 1). Euk-EH1 and Euk-EH4 recorded the smallest band number out of the total (12 and 16, respectively). Euk-EH1 had the lowest marker number of bands (46.76 KDa and 36.92 KDa) and Euk-EH4 (49.08 KDa and 9.69 KDa). Euk-EH3 had the highest number of marker protein bands (55.23 KDa, 35 KDa, 32.17 KDa, 18.78 KDa, 13.49 KDa, 12.17 KDa, 10.8 KDa, and 10.08 KDa). However, Euk-EH2 had an average number of marker protein bands (43.56 KDa, 25 KDa, 22.89 KDa, 11 KDa, and 10.34 KDa).

The polymorphism among the four dominant HAB species ranged from 70% at Euk-EH1 to 78.57% at Euk-EH4, represented by 36 polymorphic bands with an average polymorphism percentage of 75 % (Table 2). The total soluble protein according to the percentage average of band intensity in Euk-EH1 followed by Euk-EH4 was 8.335 and 6.26, respectively, that referred to the highest expression level (Table SI 2).

**Table 2:** Fraction bands number of monomorphic, polymorphic, and marker proteins, and polymorphism percentage per species in the four dominant species during red tide in Eastern Harbor

Red tide causative species	Monomorphic bands	Polymorphic bands	Total no. of bands	Unique bands	% Polymorphism
Euk-EH1	3	7	10	2	70.00
Euk-EH2	3	10	13	5	76.92
Euk-EH3	3	8	11	8	72.72
Euk-EH4	3	11	13	2	78.57
Total	12	36	48	17	
Average	3	9	12	4.25	74.55

**Fig. 3:**
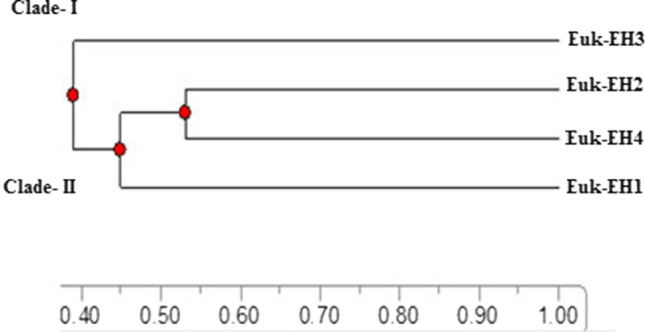
Phylogenetic dendrogram for the four dominant species during red tide in Eastern Harbor based on the total protein profiling pattern

Genetic similarity (GS) and clustering based on total protein profile pattern for the four dominant species during red tide were carried out (Fig. 3). The genetic similarly ranged from 40 to 55 %, with a mean similarity of 47.5 %. Euk-EH3 occupied a separated clade (Clade-I) with the lowest similarity among species; (40%). Species in clade-II had a similarity range of 45 to 55%. The Clade-II has diverged into two sub-clades; Euk-EH1 occupied a clade with a GS of 45%. The other sub-clade-II branched into two sub-sub-clades that comprised Euk-EH2 and Euk-EH4 with a similarity of 55%.

### Peroxidase isozymes profiling

**Fig. 4:**
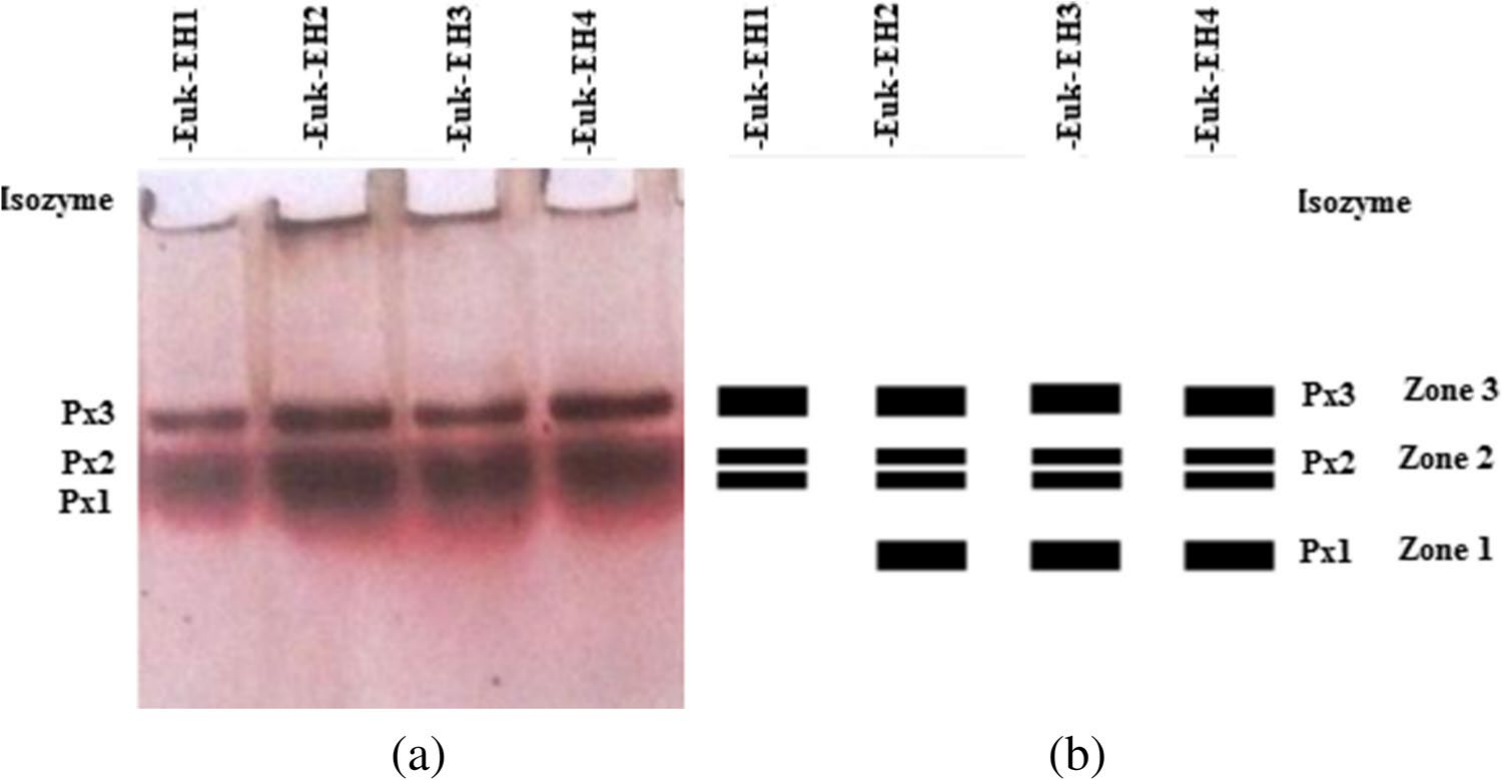
Isozyme pattern (**a**) and zymogram (**b**) of peroxidase for the four dominant species during red tide in Eastern Harbor, Px: peroxidase isozyme

The peroxidase activity showed genetic variability among the four dominant species during red tide in EH (Fig. 4a). Peroxidase zymogram showed activity as three activity zones (zone 1, zone 2, and zone 3) as was shown in Fig. 4b. These zones represented three loci responsible for the peroxidase expression in the isolates. The peroxidase banding profile resolved as a polymorphic band at zone 1 and monomorphic bands at zones 2 and 3. All isolates except Euk-EH1 showed a polymorphic band (Px1) at zone 1. The Px1 isozyme fragment expression as a single-banded monomeric homozygous enzyme. All isolates had a peroxidase activity (Px2) at zone 2 as a monomorphic double-banded pattern. The Px3 activity at zone 3 appeared as a single monomorphic band in evidence of monomerity and homozygosity.

The peroxidase banding pattern 11 flanked isoforms loci in the four isolated species. Eight loci out of 11 showed monomorphism; the other detected three loci were polymorphic, while no unique loci were detected (Table 3). The polymorphism percentage was 33.3% per species except for Euk-EH1 that had no polymorphism. The average polymorphism percentage among species was 27.27 %.

**Table 3:** Number of monomorphic/ polymorphic peroxidase fragment bands produced by three loci and polymorphism percentage per species in the four dominant species during red tide of Eastern Harbor

Red tide causative species	Monomorphic loci	Polymorphic loci	Total no. of loci	% Polymorphism
Euk-EH1	2	0	2	0
Euk-EH2	2	1	3	33.3
Euk-EH3	2	1	3	33.3
Euk-EH4	2	1	3	33.3
Total	8	3	11	
Average	2	1	2.75	27.27

The average percentage of band intensity of peroxidase that varied among species indicated its expression level (Table SI 3). DespiteEuk-EH1 did not express Px1; Euk-EH3 had the highest Px1 expression level, while Euk-EH4 had the lowest one. The heterozygous Px2 isoform reached the highest expression level at Euk-EH1 followed by Euk-EH2, Euk-EH4, and Euk-EH3. The Px3 expressed remarkably at Euk-EH4 followed by Euk-EH3, Euk-EH1, and Euk-EH2.

Peroxidase isozymes profile pattern of the four dominant species exhibited some GS (Fig. SI 1). Clustering of the isolated species based on the isozyme pattern derived a phylogenetic dendrogram. It showed the linage into two clades; Euk-EH1 with a GS of 45 % occupied Clade I.

Peroxidase isozymes profile pattern of the four dominant species exhibited some GS (Fig. SI 1). Clustering of the isolated species based on the isozyme pattern derived a phylogenetic dendrogram. It showed the linage into two clades; Euk-EH1 with a GS of 45 % occupied Clade I. Clade II diverged into two sub-clades; Euk-EH4 with GS 73% occupied the first sub-clade; the other sub-clade was shared by Euk-EH2 and Euk-EH3 whom GS was 98 %.

### DNA sequencing of 18S rRNA genes and species clustering

**Fig. 5:**
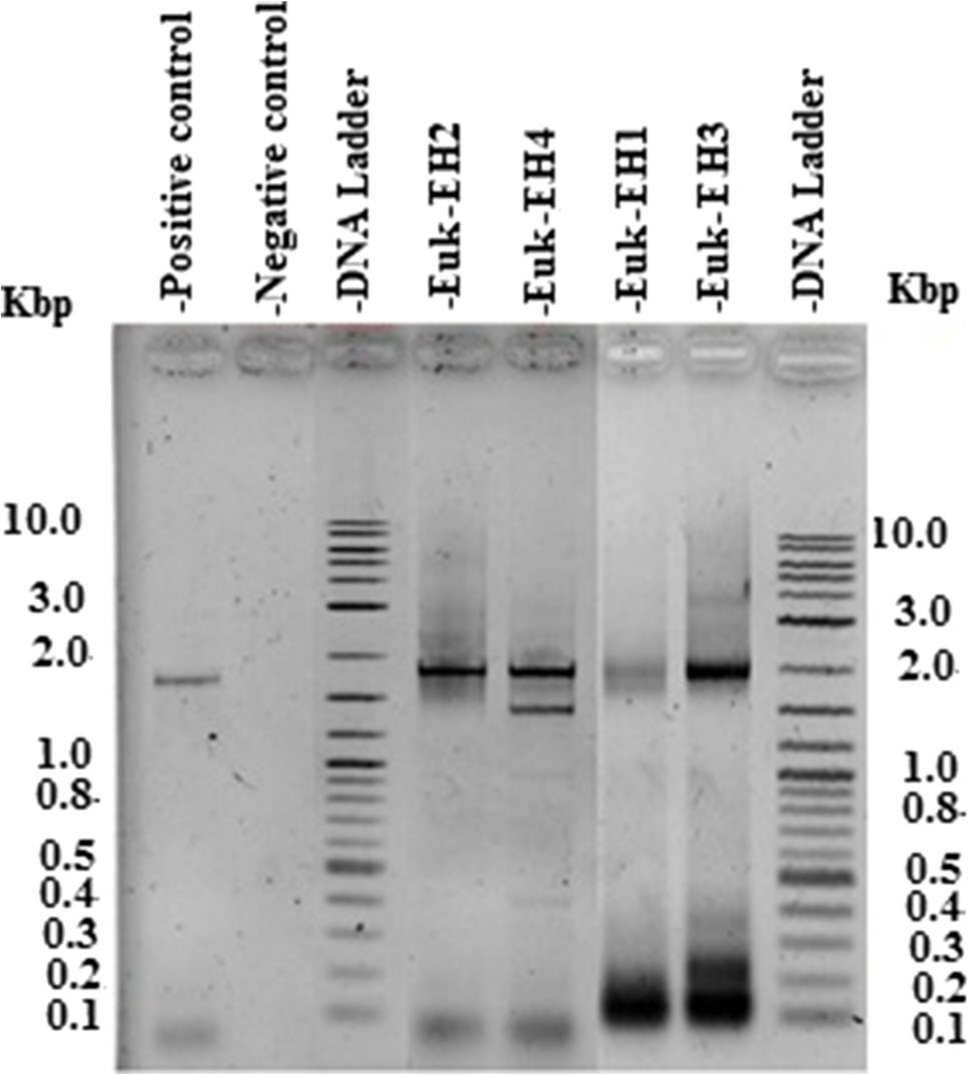
The PCR amplified product of 18S rRNA gene for the four dominant species during red tide in Eastern Harbor, using specific primers against PCR negative control (no DNA, but the water was added) and PCR positive control (DNA was added)

The extracted total genomic DNA exerted integrity against a ladder ranging from 100 bp to 10 kb (Fig. SI 2). The genomic DNA appeared as bands >10 kb in size. The size of the amplified eukaryotic 18S rRNA gene was about 1800 bp, for all studied samples (Fig. 5). Euk-EH4 revealed an amplicon of 18S rRNA gene of two rRNA gene bands; size of the upper one was 1800 bp and constituted rRNA gene amplicon. The lower band had a size of 1500 bp that may be a non-specific amplification band and that is common in PCR. The sequenced amplicon of 18S rRNA genes has deposited in Gene Bank under the accession numbers that is mentioned in Table 4.

**Table 4:** Eukaryotic 18S rRNA gene of the four dominant species during red tide isolated and cultured from Eastern Harbor of Alexandria aligned against the related sequence at gene bank for the previously identified species

	Eukaryote species	Accession numbers		Eukaryote species	Accession numbers
No.	Euk-EH1 (*Cryptophyceae* sp.)	LC377045	No.	Euk-EH2 (*Aplanochytrium* sp.)	LC377046
1	*Aplanochytrium* sp.	FJ810216	1	*Aplanochytrium* sp.	EU851171
2	*Dawsonia superba*	Y16520.1	2	*Aplanochytrium stocchinoi*	AJ519935.1
3	*E. robustum gene*	X78890.1	3	*Aplanochytrium Kerguelense*	AB022103.1
4	*S. natans*	X90413.1	4	*Oblongichytrium* sp.	AB973531.1
5	*Pirsonia formosa*	AJ561109.1	5	Uncultured *labyrinthulid*	FJ800649.1
6	*Itatiella ulei voucher Hedderson*	AY126959.1	6	Uncultured eukaryote clone	KT277637.1
7	*J. leiantha*	X91784.1	7	Uncultured *stramenopile clone*	KP685306.1
8	*Takakia lepidozioides*	AJ269686.1	8	Uncultured *Aplanochytrium clone*	KM067445.1
9	*Cryptophyceae* sp.	GQ375264.1	9	Uncultured marine eukaryote clone	AY381216.1
10	*Meiotrichum lyallii voucher Webe*	AY126960.1	10		
No.	Euk-EH3 (*Chlamydomonas* sp.)	LC377047	No.	Euk-EH4 (*Psammodictyon* sp.)	LC377048
1	*Chlamydomonas* sp.	AB972948	1	*Psammodictyon* sp.	JQ885984
2	*Chlorococcum littorale gene*	AB058336	2	*Psammodictyon panduriforme*	KU561186.1
3	*Spongiococcum tetrasporum*	KY086469	3	*Psammodictyon constrictum*	AB430617.1
4	*Tetracystis aplanospora*	KM020026.1	4	*Nitzschia dubiiformis*	AB430616.1
5	*Chlamydomonas raudensis*	JF343798.1	5	*Nitzschia commutate*	KM116105.1
6	*Chlorococcum dorsiventrale*	AB058302.1	6	*Nitzschia capitellata*	KU561195.1
7	*Chlamydomonas hedleyi*	AJ781312.1	7	Uncultured marine eukaryote	KC771178.1
8	*Chlamydomonas Concordia*	KT860848.1	8	Uncultured *stramenopile*	FN690556.1
9	*Chlorococcum minutum*	GQ122365.1	9	*Nitzschia longissima*	AY881968.1
10			10	*Bacillaria cf. paxillifer*	HM805020.1

The constructed DNA-based phylogenies revealed the similarity of the 18S RNA gene amplicon sequences of the studied samples to the known DNA sequences databases obtained from FASTA. Table 4 mentioned the known sequences with the highest similarities to the studied species. Moreover, a phylodendrogram was constructed based on similarities for each sample.

**Fig. 6:**
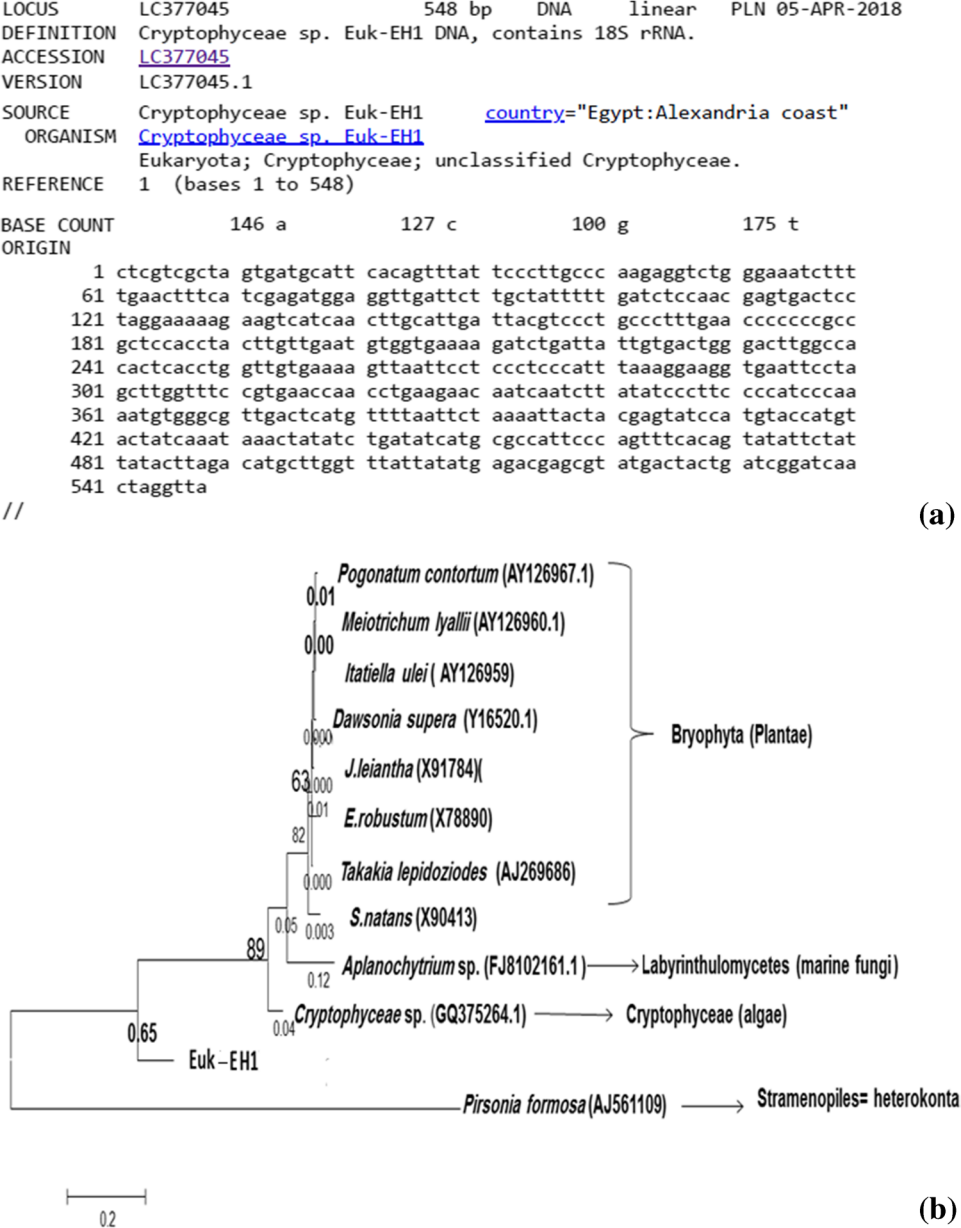
The Eastern harbor’s cultured isolated Euk-EH1 phylotype identified as *Cryptophyceae* sp. by its aligned 18S rRNA gene partial sequence of 548 bp. (**a**) which deposited under accession number LC377045 at DNA Data Bank of Japan (http://getentry.ddbj.nig.ac.jp/getentry/na/LC377045/?filetype=html). The constructed clustered consensus phylogenetic tree according to the corresponding sequences from the database (**b**), bootstrap values more than 50%, of compared algorithms are indicated at the branch roots. The bar represents 0.05 changes per nucleotide. Accession numbers of database extracted sequences are in brackets

The studied species adopted the phylogenetic dendrogram and the genetic homogeneous coefficient matrix for identification. Finally, separated phylogenetic trees were derived to reconstruct the aligned sequences with known species of the most similar one. Euk-EH1 partial sequence of 18S rDNA was 548 nt. in length (Fig. 6a), deposited in Gene Bank under the accession number LC377045. The isolate formed monophyletic lineage within the cluster of *Cryptophyceae* (Fig. 6b) with a homology of 72.4% to *Cryptophyceae* sp. that implicated a new species under Cryptophyta, subsequent with *Aplanochytrium* sp. (71.5 %) and *Pirsonia formosa* (71.4 %). Euk-EH1 possesses homogeneity with *Dawsonia superba* (71.6 %), *J. leiantha* 18S ribosomal gene of 1810 nt. (71.3%), *E. robustum* gene (71 %), *S. natans* DNA for 18S ribosomal RNA gene (70.2 %), *Itatiella ulei* voucher Hedderson (70.2%), *Takakia lepidozioides* (70.1 %), *Meiotrichum lyallii* voucher Webe (69.9 %), and *Pogonatum contortum* voucher Hedderson (69.9 %).

**Fig. 7:**
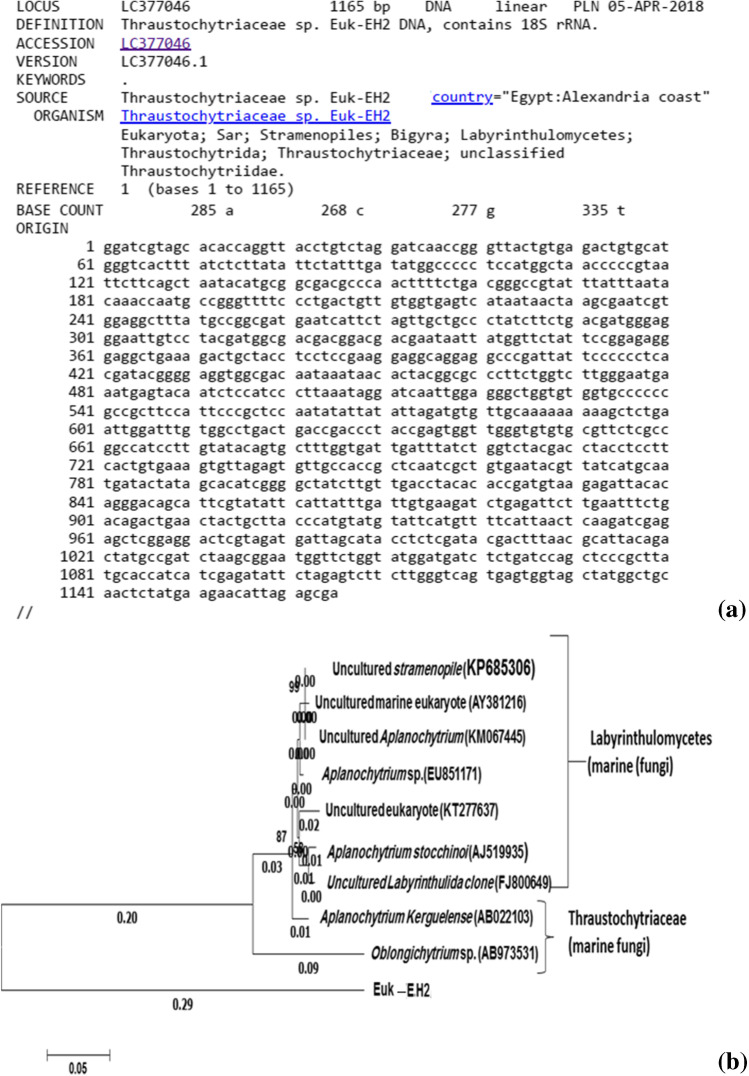
The Eastern harbor’s cultured isolated Euk-EH2 phylotype identified as *Aplanochytrium* sp. by its aligned 18S rRNA gene partial sequence of 1165 bp. (**a**) which deposited under the accession number of LC377046 at DNA Data Bank of Japan (http://getentry.ddbj.nig.ac.jp/getentry/na/LC377046/?filetype=html). The clustered as a consensus phylogenetic tree constructed according to the corresponding sequences from the database (**b**), bootstrap values more than 50%, of compared algorithms are indicated at the branch roots. The bar represents 0.05 changes per nucleotide. Accession numbers of database extracted sequences are in brackets

The Euk-EH2 partial sequence of 18S rDNA was 1165 nt. in length (Fig. 7a), deposited in Gene Bank under the accession number LC377046. The isolate formed monophyletic lineage within the cluster of Thraustochytriaceae: marine fungi (Fig. 7b.) The isolate Euk-EH2 showed a homology percentage of 73.3 % with *Oblongichytrium* sp., subsequent with *Aplanochytrium kerguelense* (74%). There was a homogeneity range between Euk-EH2 and the uncultured labyrinthulid clone (74.4%), *Aplanochytrium stocchinoi *(74.1%), the KT277637.1 uncultured eukaryote clone (73.6%),* Aplanochytrium sp.* (74.2%), the KM067445.1 uncultured *Aplanochytrium* clone (73.8%), the AY381216.1 uncultured marine eukaryote (73.8%), and the KP685306.1 uncultured stramenopile clone (73.8%).

**Fig. 8:**
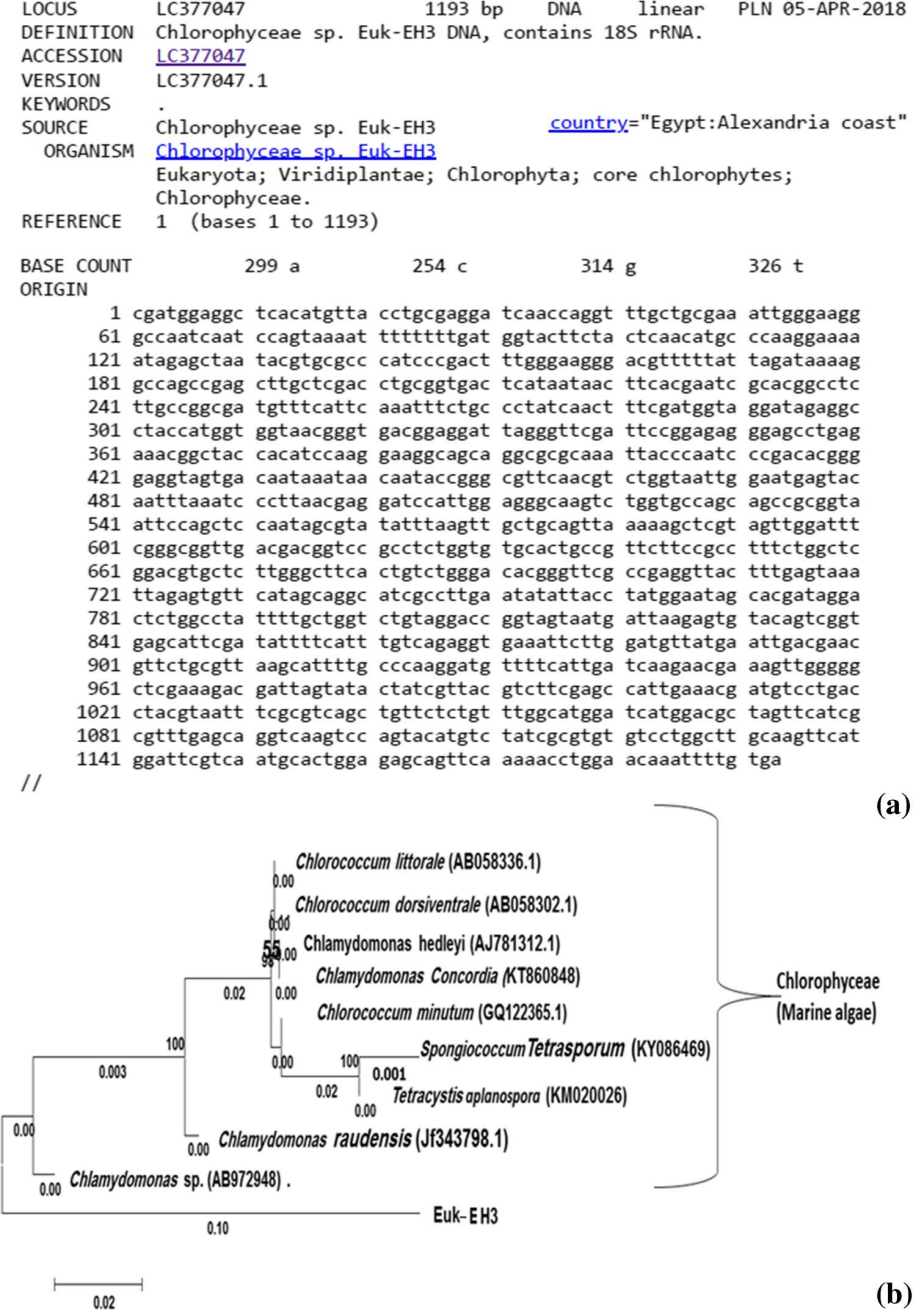
The Eastern harbor’s cultured isolated Euk-EH3 phylotype identified as *Chlamydomonas* sp. by its aligned 18S rRNA gene partial sequence of 1193 bp. (**a**) which deposited under the accession number of LC377047 at DNA Data Bank of Japan (http://getentry.ddbj.nig.ac.jp/getentry/na/LC377047/?filetype=html). The clustered as a consensus phylogenetic tree constructed according to the corresponding sequences from the database (**b**), bootstrap values more than 50%, of compared algorithms are indicated at the branch roots. The bar represents 0.02 changes per nucleotide. Accession numbers of database extracted sequences are in brackets

The Euk-EH3 identification according to its nucleotide sequence flanked as 1193 nt. (Fig. 8a). The Euk-EH3 sequence was deposited in Gene Bank under the accession number LC377047. Our isolate formed monophyletic lineage within the cluster of Chlorophyta (Fig. 8b). The isolate Euk-EH3 showed a homology percentage of 93.8 % with *Chlamydomonas* sp. and *Chlamydomonas raudensis* (92.3%), implicating new species under the genus *Chlamydomonas*, subsequent with *Tetracystis aplanospora* (90.8%) and *Spongiococcum tetrasporum* (90.7%). Euk-EH3 possess far homogeneous with *Chlorococcum minutum* (92.6%), *Chlamydomonas concordia* (92.3%), *Chlamydomonas hedleyi* (91.6%), *Chlorococcum dorsiventrale* (91.7%), and *Chlorococcum littorale* (91.7%).

**Fig. 9:**
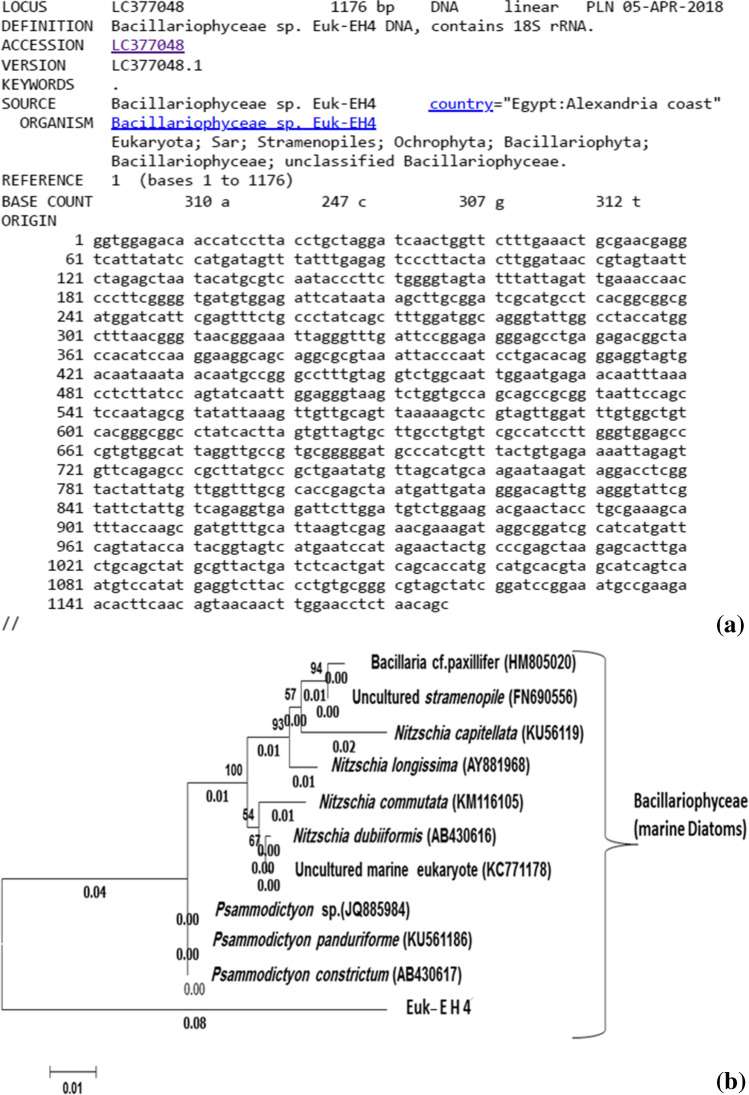
The Eastern harbor’s cultured isolated Euk-EH4 phylotype identified as *Psammodictyon* sp. by its aligned 18S rRNA gene partial sequence of 1176 bp. (**a**) which deposited under accession number LC377048 at DNA Data Bank of Japan (http://getentry.ddbj.nig.ac.jp/getentry/na/LC377048/?filetype=html). The clustered as a consensus phylogenetic tree constructed according to the corresponding sequences from the database (**b**), bootstrap values more than 50%, of compared algorithms are indicated at the branch roots. The bar represents 0.04 changes per nucleotide. Accession numbers of database extracted sequences are in brackets

The Euk-EH4 partial sequence of 1176 nucleotide was identified (Fig. 9a) and deposited in Gene Bank under the accession number LC377048. The isolate formed monophyletic lineage within the cluster of Bacillariophyta (Fig. 9b). The isolate Euk-EH4 showed homology parentage of 95.1% with *Psammodictyon constrictum* and *Psammodictyon panduriforme*, subsequent with *Psammodictyon* sp. (94.7%) implicating new species under the genus *Psammodictyon constrictum* (Krayesky et al. 2009; Lobban 2015). The isolate Euk-EH4 showed homology percentage to uncultured marine (93.2%), *Nitzschia dubiiformis* (93.4%), *Nitzschia commutata* (93.2%), *Nitzschia longissima* (92.7%), *Nitzschia capitellata* (92.5%), uncultured *strameno* (92.5%), and *Bacillaria cf. paxillifer* (92.2%). Separated phylogenetic trees were derived to reconstruct the previous phylotypes against the most similar known species in the gene bank. The genotype Euk-EH2 species were morphologically identified as *chrysophyceaen* species, *Ochromonas* sp., but the aligned 18S rDNA sequences proved Euk-EH2 identification as *Aplanochytrium sp*., belonging to class Labyrinthulomycetes, which used to belong to the defunct fungal phylum Labyrinthulomycota.

## Discussion

The occurrence of HAB in the Alexandria region is frequent almost the summer reaches a maximum at early August to half of September. This may be due to germination of the sink cysts in the harbor bottom or navigational movement and water streams. HABs are frequent in Alexandria and previously were isolated from the Eastern harbor (EH) and identified morphologically. The recent study is unique because it inquired about the reliability of morphological identification for the isolated HABs from EH using biochemical and molecular technologies. The genetic variations detection solved the potential collapses among HAB morpho-species rather than morphological identification.

### Polymorphism and phylogenetic relations based on protein profile

The HABs causative involves different species from various taxonomical levels. Polymorphisms among species enable some species to be dominant according to their genomic entity for modification and adaptation to stand with selection forces. Polymorphism can contribute to the characterization of different species depending on genetic variability inter- and intra-species that enable acclimation to different habitats (Hugall and Stuart-Fox 2012). The genetic variation could characterize modulation of the adaptive biomolecules, protein, and isoenzymes (Rouxel et al. 2001; Saini and Yadav 2014). Protein profile enabled this study to detect genetic variation among the studied species. We documented specific marker protein bands for each isolate, two bands in both Euk-EH1 and Euk-EH4, and five bands for Euk-EH2 and 8 for Euk-EH3. In addition, the protein pattern showed a 75% average polymorphism among our studied species. These results pointed out the genetic variations among species due to the expressive entity of protein for the gene sequence of an organism. The result agreed with the routine usage of protein profiling for detecting variability within, among species, and populations depending on polymorphism (Singh et al. 2017; Sihmar et al. 2020). Protein profile variability can determine differences among species such as between cultured and endosymbiotic species like in *Symbiodinium* sp. (Stochaj and Grossman 2008). The efficiency of using protein profile or characterization of our species agreed with the result of Chan et al. (2002 and 2004) regarding using PAGE analysis to differentiate between taxonomically confused strains of a single species as a powerful taxonomic tool.

The variation in band intensity showed that protein differed qualitatively and quantitatively among species. This result agreed with those previous studies that reported not only differences in the expression and assembly of the available protein in algae (Mayfield et al. 2003; Wang et al. 2014; Shi et al. 2021) but also de novo synthesized proteins in unicellular green-alga-like *Scenedesmus phlyctidium* upon external applications (Nedeva et al. 2008). So we suggest that qualitative and quantitative variation in band intensity among species can indicate taxonomy and the potentiality of toxicity level upon expression level in toxic species, respectively. This suggestion agreed with the previous studies that pointed out using proteomics analysis for detecting toxicity in HABs (Jiang et al. 2015; Li et al.; 2019; and Zingone et al. 2021) to investigate the genetic basis for the production of toxins and allelochemicals. ability (Anderson et al. 2012). The GS based on the protein polymorphism pointed to a close relation between Euk-EH4 (*Psammodictyon constrictum*) and Euk-EH2 (*Aplanochytrium sp.*), subsequent by Euk-EH1 (*Cryptophycean sp.*) while Euk-EH3 (*Chlamydomonas raudensis*) had a loose relation due to its unique position in a separate cluster. This result agreed with a previous study that related thraustochytrids (*Aplanochytrium* sp.) to heterokont algae such as the chrysophytes and diatoms (Cavalier-Smith 1993; Bongiorni et al*.* 2005). Moreover, thraustochytrids are phylogenetically close to the heterokont algae (e.g., diatoms and brown algae) and cited as microalgae (Byreddy et al. 2016). In addition, thraustochytrids and *Prorocentrum* were involved in a separated cluster at a phylogenetic dendrogram with a similarity percentage of 99 % (Caamaño et al. 2017).

The recent study has used molecular identification for HBAs according to referring by Hallegraeff (1993), Hallegraeff et al. (2003), and Anderson et al. (2012). Anderson reported DNA probing and biochemical methods in HABs monitoring and taxonomy, e.g., dinoflagellates, haptophytes, diatoms, raphidophytes, cyanobacteria, and cysts. Also, Liu has reported phylogenetic analyses and comparative genomic analyses of coastal diatoms (*Skeletonema* species) using full-length mitochondrial genomes (Liu et al. 2021a) and chloroplast genome (Liu et al. 2021b).

### Polymorphism and phylogenetic relations based on peroxidase isozymes

Peroxidases include volatile organohalogens, biosynthesized by marine organisms to oxidize halide ions by using hydrogen (Neidleman and Geigert 1986; Wever et al. 1991). Peroxidase isozymes profiling is expressive for the genetic variation since alloenzymes are codominant markers (Saini and Yadav 2014). We suggested peroxidase coding as a protein product of two loci in Euk-EH1 and three loci in the other studied species (Px1, Px2, and Px3). This suggestion was endorsed by detected polymorphism among species for locus-1 where there was no activity for Px1 isozyme at zone 1 in Euk-EH1. Locus 2 and locus 3 of peroxidase did not show any polymorphism among isolates. Expression of Px1 in Euk-EH2, Euk-EH3, and Euk-EH4 as a single band suggests a monomeric homozygous isoenzyme entity coded by locus 1**.** The double-banded of Px2 suggested the monomer entity and heterozygosity of peroxidase at the Px2 locus (Micales et al. 1992). The single band at locus 3 suggested the monomeric and homozygous entity of Px3. This result agreed with similar literature that reported the monomeric nature of the enzyme with two allelic variants in some fungi (Micales et al. 1992). Our results agreed with these studies on *Salvadora oleoides* that pointed to the peroxidase monomer entity, expressed by five loci of variant alleles (Saini and Yadav 2014). Peroxidase phyletic dendrogram cleared the high similarity, sharing the same sub-cluster, of *Aplanochytrium* sp. (Euk-EH2) and *Chlamydomonas raudensis* (Euk-EH3). Both Euk-EH2 and Euk-EH3 were close to *Psammodictyon constrictum* Euk-EH4 than *Cryptophycean sp.* Euk-EH1 segregated on a separated cluster as loosely linked species. This agreed with relating thraustochytrids (including *Aplanochytrium sp.* or Euk-EH2) to heterokont algae such as the chrysophytes and diatoms (including *Psammodictyon constrictum* or Euk-EH4) in some taxonomical studies (Cavalier-Smith 1993; Bongiorni et al. 2005).

Cluster species upon GS derived from peroxidase isozymes is efficient in detecting the genetic diversity due to the following: alleles variability (Schaal et al. 1991; Weir 1996; Saini and Yadav 2014), codominant expression, and absence of epistasis (Saini and Yadav 2014). Isozyme loci efficiency in characterization and description attributed to the biochemical genetic variation and population genetics (Saini and Yadav 2014).

Cluster species upon GS derived from peroxidase isozymes is efficient in detecting the genetic diversity due to the following: allele’s variability (Schaal et al. 1991; Weir 1996; Saini and Yadav 2014), codominant expression, and absence of epistasis (Saini and Yadav 2014). The biochemical genetic variation and population genetics are the axes of isozyme loci efficiency in characterization and description (Saini and Yadav 2014). Furthermore, the characterization potentiality of isozymes is high in deterring intra-and inter-specific variations among species (Medhabati et al. 2013; Saini and Yadav 2013).

This study showed a varied expression for peroxidase loci among species as indicated by band intensity. This expression suggested that the dominance of the studied species over the others may be due to the sustainable defense system trigging by peroxidases. This trigging is high in Euk-EH2 and Euk-EH4 whom habits are epiphytic free-living, parasitic, or symbiont on some marine living organisms. These results go parallel with some studies on phytoplankton of Antarctic lake (Neale and Priscu 1995): aplanochytrids (Damare and Raghakumarb, 2006), *Scenedesmus phlyctidium* (Nedeva et al. 2008), and *Chlamydomonas raudensis* (Dolhi et al. 2013; Stahl 2014). These studies reported the induction of stress-related genes of peroxidase isozymes in some species for contribution in supporting phytoplankton acclimation in response to constitutive, long-term environmental stress. Our results agreed with peroxidase nature as scavenger’s enzymes and defense system triggers (Schaffer and Bronnikova 2012). Moreover, peroxidase has a role in the ATP requirements maintenance in *Chlamydomonas raudensis* (Dolhi et al. 2013) and psychrophilic and mesophilic alga (Morgan-Kiss et al. 2002). The role of peroxidase in physiological processes in the biosynthesis of lignin and hormone is significant (Kirk and Farrell 1987; Shigeoka et al. 1980).

### Specie identification by18S rRNA gene operon sequences

The four sequenced samples were identified upon alignment with the available sequence of DNA database for 18S rRNA (http://www.ebi.ac.uk/Tools/sss/fasta/). The Euk-EH1, Euk-EH2, Euk-EH3, and Euk-EH4 were identified as *Cryptophycean* sp., *Aplanochytrium* sp., *Chlamydomonas raudensis*, and *Psammodictyon constrictum* and deposited in the international DNA database of the Bank of Japan under accession numbers of LC377045, LC377046, LC377047, and LC377048, respectively.

The species Euk-EH2 was previously recorded in the harbor among the unidentified microflagellates where it contributed 10.4 % of the total standing crop during 2000 (Mikhail 2001), forming several blooms in combination with *P*. *minimum* and *Chattonella antiqua* during May 2001 (Mikhail 2003), with *S*. *costatum* in May 2004 (Mikhail et al. 2005) and September 2007 (Mikhail and Halim 2009). Interestingly, this study identified the genotype Euk-EH2 species morphologically as chrysophyceaen species; *Ochromonas* sp. but the applied molecular taxonomy based on DNA sequences had identified Euk-EH2 as *Aplanochytrium* sp., belonging to class Labyrinthulomycetes, which used to belong to the defunct fungal phylum Labyrinthulomycota.

Our result agreed with the citations that genetic differences are sufficient to separate HABs species, even when these assignments differ from those that are morphologically identified (Anderson et al. 2012). The results of using 18S rRNA gene operon sequences in our study were in harmony with the results of Khaw et al. (2020) that reported using 18S rDNA primers as a simple method for cultured eukaryotic microalgae identification. Using 18S rRNA in our results was in harmony with the results of Khaw et al. (2020) that reported using 18S rDNA primers as a simple method for cultured eukaryotic microalgae identification. Phylogenetic reconstruction of relationships among HAB taxa are preferably predicted based on sequence analysis of one or a few genes, typically including the ribosomal ITS, cytochrome-c oxidase subunit 1 (cox1) genes, LSU rDNA, SSU rDNA, and ISH (Pocock et al. 2004; O’Halloran et al. 2006; Auinger et al. 2008; Hong et al. 2008; Mortazavi et al. 2008; Damare and Raghukumar 2010; Anderson et al. 2012; Fariman and Javid 2013; Dierssen et al. 2015).

Sequence alignment of 18S rDNA pointed agreed with the total protein profiling pattern regarding determining the relationship between species. Protein profiling referred to the close relation between *Aplanochytrium* sp., Euk-EH2, and *Psammodictyon* sp., Euk-EH4. This agreed with the phylogenetic taxonomy of thraustochytrids (*Aplanochytrium* sp.) as heterokont-related algae such as the chrysophytes and diatoms (Cavalier-Smith 1993; Bongiorni et al. 2005).

This study informed that molecular characterization is definitively more than the morphological identification of microalgae, especially HAB species. This followed methodology agreed with Anderson et al.’s (2012) report for using DNA probing, molecular data, and phylogenetic analysis for accurate species interpretation concept including strain variation in HABs to understand and manage these phenomena. The study declared the collapse of *Aplanochytrium sp.* which was morphologically identified as the chrysophyceaen species: *Ochromonas* sp. before a decisive molecular classification. The study offered a genetic database based on the dominant HAB species at EH for supporting phenomena management to avoid any threats to marine life or human health. The study knocked alarm for discoloration incidence in the harbor despite detecting neither fish kill nor human health problems during these blooms. If any increase in the potential harmful species number and blooms magnification in response to rapidly changing environmental conditions have occurred, the harbor will be at risk. However, biogenetics and molecular identification of some red tide species in Alexandria waters were carried out for the first time, and it may be one from the pioneer Egyptian studies. It is a new valuable addition to the efforts previously done on bloom events in the harbor. Our effort in molecular identification of HBAs causative species is going on with the recommendation of Anderson et al. (2021). Anderson reported that efforts are needed to expand and sustain the data collection regionally and nationally to save accurate information on the scale and nature of the problem in HBAs monitoring due to facing HBAs diversity.

## Conclusion

This study provided a molecular database for the most dominant red tide constituents in EH at Alexandria region, north Egypt, on the Mediterranean Sea. The study adopted biochemical and molecular markers that included protein and isozyme profiling, 18S rRNA gene operon sequencing, and clustering. This work concluded that the dominant causative species during red tide in EH were *Aplanochytrium* sp., *Chlamydomonas* sp., *Cryptophyceae* sp., and *Psammodictyon* sp. depending on sequence analysis of 18S rRNA that we deposited at gene bank under accession numbers of LC377045, LC377046, LC377047, and LC377048, respectively. The current work pointed out the effectiveness of protein and isozymes profiling as probing methods for inquiring about genetic variation among species. Profiling patterns succeed in classifying isolate cultures into four species and clustering *Cryptophyceae* sp. on as separated clade. Protein pattern recognized 17 marker bands specific to species where *Chlamydomonas* sp. possessed eight bands out of them. Peroxidase pattern revealed expression with three loci in all species except *Cryptophyceae* sp. that own only two loci. Identification of *Aplanochytrium* sp. during red tide is surprising due to the fungal entity; belonging to class Labyrinthulomycetes, phylum Labyrinthulomycota for defunct fungi. Our molecular identification revealed the entity of red tide constituents in EH that may contribute to the protective measures for restricting the harmful effects of the phenomenon.

## Supplementary information

Fig. S1 (PNG 79 kb)

High resolution image (TIF 52 kb)

Fig. S2 (PNG 1312 kb)

High resolution image (TIF 94 kb)

Table S1 (DOCX 50 kb)

Table S2 (DOCX 52 kb)

Table S3 (DOCX 46 kb)

## Data Availability

All data used to conduct this study is provided within the manuscript and as supplementary material.
