# Supplementary figures and images for: Molecular taxonomical identification and phylogenetic relationships of some marine dominant algal species during red tide and harmful algal blooms along Egyptian coasts in the Alexandria region
Source: Environ Sci Pollut Res Int. 2022 Mar 14;29(35):53403–19. doi: 10.1007/s11356-022-19217-8 (PMC9343293; doi:10.1007/s11356-022-19217-8)

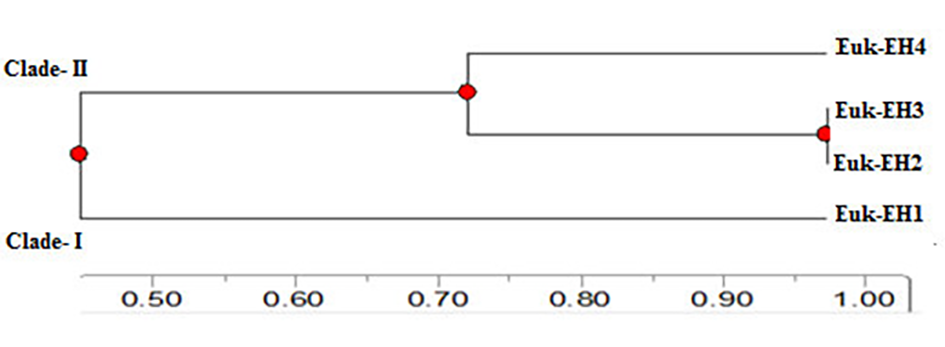

Supplement: Supplementary file 1 — (PNG 79 kb) [file 11356_2022_19217_Fig10_ESM.png]

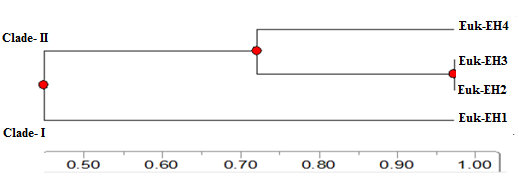

Supplement: Supplementary file 2 — High resolution image (TIF 52 kb) [file 11356_2022_19217_MOESM1_ESM.tif]

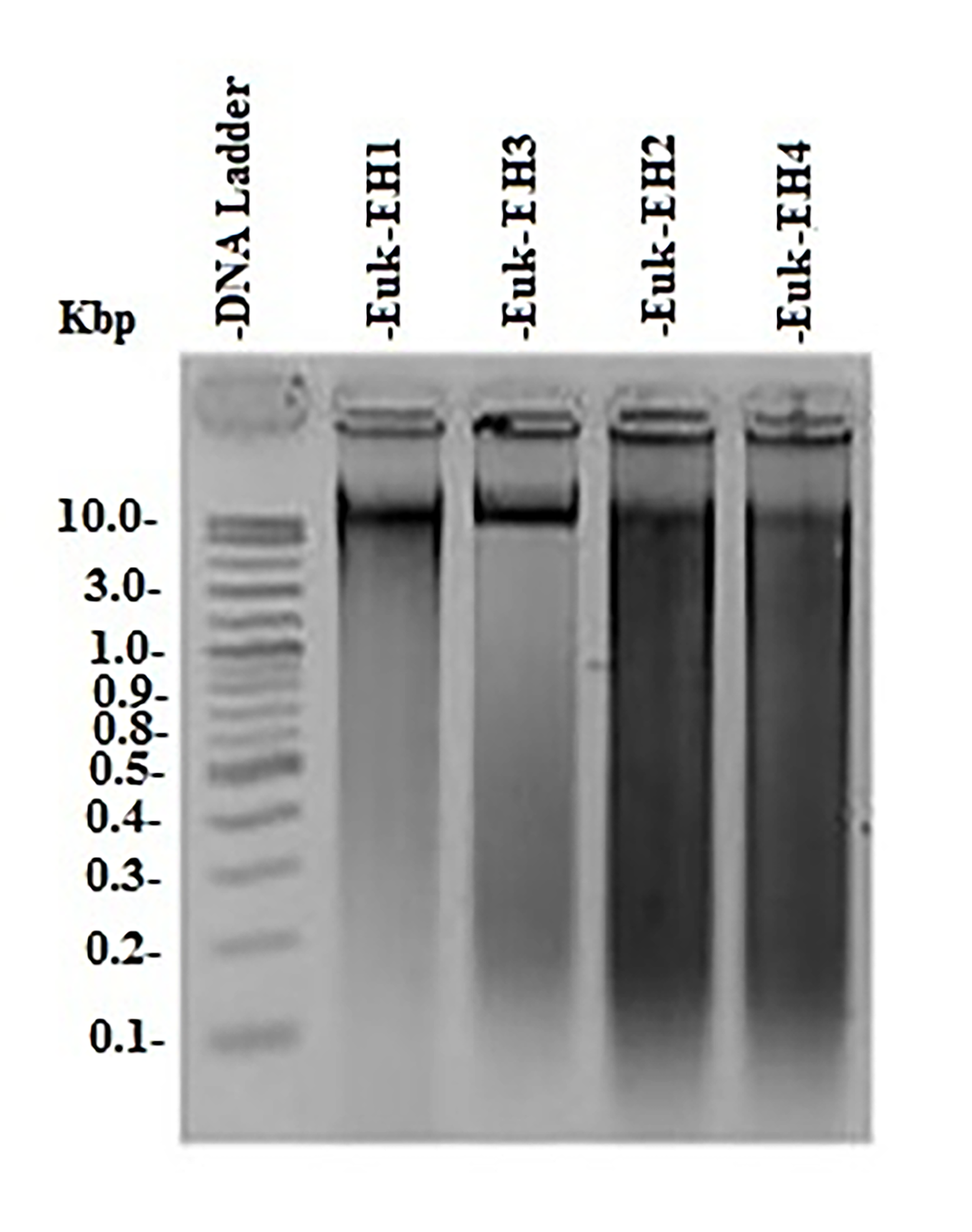

Supplement: Supplementary file 3 — (PNG 1312 kb) [file 11356_2022_19217_Fig11_ESM.png]

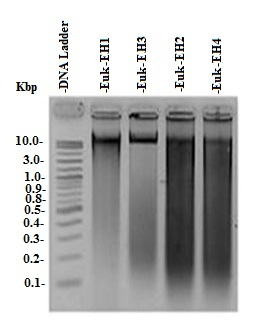

Supplement: Supplementary file 4 — High resolution image (TIF 94 kb) [file 11356_2022_19217_MOESM2_ESM.tif]
