# Supplementary material for: Molecular taxonomical identification and phylogenetic relationships of some marine dominant algal species during red tide and harmful algal blooms along Egyptian coasts in the Alexandria region
Source: Environ Sci Pollut Res Int. 2022 Mar 14;29(35):53403–19. doi: 10.1007/s11356-022-19217-8 (PMC9343293; doi:10.1007/s11356-022-19217-8)
Supplement: Supplementary file 5 — (DOCX 50 kb) [file 11356_2022_19217_MOESM3_ESM.docx]

| Table S1 Presence and absence of protein fractions for the four dominant species during red tide in Eastern Harbor | | | | | |
| --- | --- | --- | --- | --- | --- |
| **Band no.** | **Protein Marker (KDa)** | **Euk-EH1** | **Euk-EH2** | **Euk-EH3** | **Euk-EH4** |
| **1** | **121.534** | 0 | 0 | 1 | 1 |
| **2** | **110.245** | 1 | 0 | 1 | 0 |
| **3** | **101.257** | 1 | 1 | 0 | 0 |
| **4** | **92.794** | 1 | 0 | 1 | 1 |
| **5** | **88.190** | 1 | 1 | 0 | 0 |
| **6** | **73.375** | 1 | 1 | 1 | 1 |
| **7** | **59.995** | 1 | 1 | 1 | 1 |
| **8** | **55.229** | 0 | 0 | 1 | 0 |
| **9** | **52.326** | 1 | 1 | 0 | 0 |
| **10** | **49.080** | 0 | 0 | 0 | 1 |
| **11** | **46.758** | 1 | 0 | 0 | 0 |
| **12** | **45.000** | 0 | 1 | 1 | 0 |
| **13** | **43.559** | 0 | 1 | 0 | 0 |
| **14** | **41.815** | 0 | 1 | 1 | 1 |
| **15** | **40.194** | 0 | 0 | 1 | 1 |
| **16** | **38.238** | 0 | 1 | 0 | 1 |
| **17** | **36.917** | 1 | 0 | 0 | 0 |
| **18** | **35.000** | 0 | 0 | 1 | 0 |
| **19** | **33.434** | 1 | 1 | 0 | 1 |
| **20** | **32.165** | 0 | 0 | 1 | 0 |
| **21** | **27.089** | 0 | 0 | 1 | 1 |
| **22** | **25.000** | 0 | 1 | 0 | 0 |
| **23** | **24.159** | 1 | 0 | 0 | 1 |
| **24** | **22.890** | 0 | 1 | 0 | 0 |
| **25** | **20.111** | 0 | 1 | 1 | 1 |
| **26** | **18.783** | 0 | 0 | 1 | 0 |
| **27** | **16.411** | 1 | 1 | 1 | 1 |
| **28** | **15.446** | 0 | 1 | 0 | 1 |
| **29** | **13.485** | 0 | 0 | 1 | 0 |
| **30** | **12.171** | 0 | 0 | 1 | 0 |
| **31** | **11.003** | 0 | 1 | 0 | 0 |
| **32** | **10.798** | 0 | 0 | 1 | 0 |
| **33** | **10.607** | 0 | 1 | 0 | 1 |
| **34** | **10.338** | 0 | 1 | 0 | 0 |
| **35** | **10.084** | 0 | 0 | 1 | 0 |
| **36** | **9.697** | 0 | 0 | 0 | 1 |
| **Total= 65** |  | 12 | 18 | 19 | 16 |
