# Supplementary material for: Molecular taxonomical identification and phylogenetic relationships of some marine dominant algal species during red tide and harmful algal blooms along Egyptian coasts in the Alexandria region
Source: Environ Sci Pollut Res Int. 2022 Mar 14;29(35):53403–19. doi: 10.1007/s11356-022-19217-8 (PMC9343293; doi:10.1007/s11356-022-19217-8)
Supplement: Supplementary file 6 — (DOCX 52 kb) [file 11356_2022_19217_MOESM4_ESM.docx]

| Table S2 Percentage of band intensity as an indicator to the protein expression level in for the four dominant species during red tide in Eastern Harbor | | | | | |
| --- | --- | --- | --- | --- | --- |
| **Band no.** | **Protein Marker (KDa)** | **Euk-EH1** | **Euk-EH2** | **Euk-EH3** | **Euk-EH4** |
| **1** | **121.534** | 0 | 0 | 1.18 | 3.01 |
| **2** | **110.245** | 3.20 | 0 | 9.64 | 0 |
| **3** | **101.257** | 4.98 | 4.38 | 0 | 0 |
| **4** | **92.794** | 5.82 | 0 | 1.84 | 3.47 |
| **5** | **88.190** | 14.59 | 1.82 | 0 | 0 |
| **6** | **73.375** | 6.23 | 1.78 | 5.05 | 12.72 |
| **7** | **59.995** | 7.26 | 10.37 | 1.33 | 3.59 |
| **8** | **55.229** | 0 | 0 | 3.55 | 0 |
| **9** | **52.326** | 15.03 | 3.10 | 0 | 0 |
| **10** | **49.080** | 0 | 0 | 0 | 11.51 |
| **11** | **46.758** | 5.68 | 0 | 0 | 0 |
| **12** | **45.000** | 0 | 14.37 | 2.21 | 0 |
| **13** | **43.559** | 0 | 2.09 | 0 | 0 |
| **14** | **41.815** | 0 | 2.71 | 3.73 | 17.76 |
| **15** | **40.194** | 0 | 0 | 3.38 | 2.81 |
| **16** | **38.238** | 0 | 5.56 | 0 | 10.02 |
| **17** | **36.917** | 9.26 | 0 | 0 | 0 |
| **18** | **35.000** | 0 | 0 | 2.53 | 0 |
| **19** | **33.434** | 9.44 | 7.17 | 0 | 8.26 |
| **20** | **32.165** | 0 | 0 | 10.73 | 0 |
| **21** | **27.089** | 0 | 0 | 6.20 | 9.89 |
| **22** | **25.000** | 0 | 10.41 | 0 | 0 |
| **23** | **24.159** | 11.70 | 0 | 0 | 5.52 |
| **24** | **22.890** | 0 | 1.65 | 0 | 0 |
| **25** | **20.111** | 0 | 6.41 | 5.61 | 4.06 |
| **26** | **18.783** | 0 | 0 | 9.56 | 0 |
| **27** | **16.411** | 6.81 | 3.04 | 6.89 | 3.82 |
| **28** | **15.446** | 0 | 5.59 | 0 | 0.65 |
| **29** | **13.485** | 0 | 0 | 7.92 | 0 |
| **30** | **12.171** | 0 | 0 | 3.63 | 0 |
| **31** | **11.003** | 0 | 9.92 | 0 | 0 |
| **32** | **10.798** | 0 | 0 | 14.30 | 0 |
| **33** | **10.607** | 0 | 4.27 | 0 | 0.26 |
| **34** | **10.338** | 0 | 5.36 | 0 | 0 |
| **35** | **10.084** | 0 | 0 | 0.73 | 0 |
| **36** | **9.697** | 0 | 0 | 0 | 2.66 |
| **Total= 65** |  | 100 | 100 | 100 | 100 |
| **Average** | | 8.335 | 5.555 | 5.264 | 6.251 |
