# Supplementary material for: Molecular taxonomical identification and phylogenetic relationships of some marine dominant algal species during red tide and harmful algal blooms along Egyptian coasts in the Alexandria region
Source: Environ Sci Pollut Res Int. 2022 Mar 14;29(35):53403–19. doi: 10.1007/s11356-022-19217-8 (PMC9343293; doi:10.1007/s11356-022-19217-8)
Supplement: Supplementary file 7 — (DOCX 46 kb) [file 11356_2022_19217_MOESM5_ESM.docx]

| **Table S3** Percentage of band intensity as an indicator to peroxidase isoforms expression level in for the four dominant species during red tide in Eastern Harbor | | | | | | |
| --- | --- | --- | --- | --- | --- | --- |
| **Isoform no.** | **RF.** | **Band Intensity** | | | | |
|  |  | **Euk-EH1** | **Euk-EH2** | | **Euk-EH3** | **Euk-EH4** |
| **Px 3** | 0.420 | 26.36 | | 24.04 | 30.75 | 37.32 |
| **Px 2** | 0.517 | 30.77 | | 18.59 | 17.44 | 21.06 |
|  | 0.555 | 42.87 | | 44.08 | 33.43 | 31.96 |
| **Px 1** | 0.632 | ------ | | 13.28 | 18.38 | 9.66 |
| **Total** |  | 100 | | 100 | 100 | 100 |
| Px: Peroxidase isoform, RF = Relative distance migrated by the band | | | | | | |
